# QUBO Problem Formulation of Fragment-Based Protein–Ligand Flexible Docking

**DOI:** 10.3390/e26050397

**Published:** 2024-04-30

**Authors:** Keisuke Yanagisawa, Takuya Fujie, Kazuki Takabatake, Yutaka Akiyama

**Affiliations:** 1 Department of Computer Science, School of Computing, Tokyo Institute of Technology, Meguro-ku, Tokyo 152-8550, Japan; 2 Middle Molecule IT-Based Drug Discovery Laboratory (MIDL), Tokyo Institute of Technology, Meguro-ku, Tokyo 152-8550, Japan; 3 Ahead Biocomputing, Co., Ltd., Kawasaki-shi 210-0007, Kanagawa, Japan; fujie@ahead-biocomputing.co.jp; 4 Toshiba Digital Solutions Corporation, Kawasaki-shi 212-8585, Kanagawa, Japan; kazuki1.takabatake@toshiba.co.jp

**Keywords:** protein–ligand docking, flexible docking, compound fragment, combinatorial optimization, quantum annealing, simulated quantum annealer, quadratic unconstrained binary optimization (QUBO), SQBM+

## Abstract

Protein–ligand docking plays a significant role in structure-based drug discovery. This methodology aims to estimate the binding mode and binding free energy between the drug-targeted protein and candidate chemical compounds, utilizing protein tertiary structure information. Reformulation of this docking as a quadratic unconstrained binary optimization (QUBO) problem to obtain solutions via quantum annealing has been attempted. However, previous studies did not consider the internal degrees of freedom of the compound that is mandatory and essential. In this study, we formulated fragment-based protein–ligand flexible docking, considering the internal degrees of freedom of the compound by focusing on fragments (rigid chemical substructures of compounds) as a QUBO problem. We introduced four factors essential for fragment–based docking in the Hamiltonian: (1) interaction energy between the target protein and each fragment, (2) clashes between fragments, (3) covalent bonds between fragments, and (4) the constraint that each fragment of the compound is selected for a single placement. We also implemented a proof-of-concept system and conducted redocking for the protein–compound complex structure of Aldose reductase (a drug target protein) using SQBM+, which is a simulated quantum annealer. The predicted binding pose reconstructed from the best solution was near-native (RMSD = 1.26 Å), which can be further improved (RMSD = 0.27 Å) using conventional energy minimization. The results indicate the validity of our QUBO problem formulation.

## 1. Introduction

Computational methods have been widely employed in the field of drug discovery. Structure-based drug discovery (SBDD) involves exploring potential drug candidate compounds using tertiary structures of a target protein. Unlike ligand-based drug discovery, which relies on characteristics of known active compounds, structure-based drug discovery can find drug candidates with novel scaffolds.

Protein–ligand docking is widely used in SBDD. This calculation estimates the binding pose between the protein and the chemical compound (as a candidate for strongly interacting ligand) with their estimated binding free energy. The performance of the docking calculation depends on (1) the effectiveness of the binding pose search algorithm and (2) the accuracy of the scoring function used to estimate the binding free energy. Various docking tools have been proposed over many years [1,2,3,4]. In particular, the search for compound binding poses must consider numerous internal degrees of freedom of the compound as well as its translation and rotation. However, exhaustive exploration of binding poses (including translation, rotation, and internal degree of freedom) cannot be handled in a realistic amount of time, and various heuristic strategies are employed. For instance, AutoDock 4, developed at the Scripps Research institute, utilizes the Lamarckian Genetic Algorithm [5]. AutoDock Vina was developed as a successor to AutoDock and estimates binding poses using an iterated local search optimizer that conducts local optimization and compound conformation mutation repeatedly [6,7]. Additionally, Glide is a widely used commercial tool that performs an exhaustive search while gradually excluding unpromising binding pose candidates by several filters (such as site-point search, diameter test, and subset test) [8,9].

Another binding pose search strategy is fragment-based docking, wherein the compound is decomposed into fragments (chemical substructures which have no internal degrees of freedom), and the binding poses of the compounds are reconstructed after separately processing each fragment. For example, FlexX [10] utilizes an incremental construction algorithm placing the first fragment in the protein pocket and extending new fragments from it. REstretto [11] utilizes relative positions of fragments with enumerating feasible compound conformers, resulting in rapid and comprehensive exploration. eHiTS [12] constructs a graph representing covalent bonds and clashes between a vast number of candidate fragment placements. It subsequently performs maximum clique finding to obtain combinations of fragment placements that form consistent compound structures. Decomposing compounds into fragments has a significant advantage that has fewer unique fragments owing to the commonality of fragments across compounds, leading to a faster calculation. Zsoldos et al. [12] reported four times faster calculation with the reuse of the intermediate results. This approach is promising given the recent exponential growth in compound library size; for example, the number of compounds in the ZINC database was approximately 34 million in 2012 [13] and 1.4 billion in 2020 [14].

The strategy of eHiTS involves transforming docking calculations to a combinatorial optimization problem to find a set of fragment placements which is consistent as a compound structure through maximum clique finding. While many important combinatorial optimization problems are NP-hard and difficult to solve, efforts were recently made to find optimal solutions for NP-hard combinatorial optimization problems rapidly using an Ising machine after mapping the problem to an Ising model [15]. Various implementation approaches are proposed for the Ising machine, such as D-Wave Systems Inc.’s quantum computer based on quantum annealing [16,17], NTT research Inc.’s Coherent Ising Machine (an optical computer) [18], and implementations on classical computers powered by graphics processing units (GPUs) and field-programmable gate arrays (FPGAs), such as NEC’s vector annealing, Fujitsu’s Digital Annealer [19], and Toshiba Digital Solutions Corporation’s SQBM+, which is based on the simulated bifurcation algorithm (SB algorithm) [20].

The quadratic unconstrained binary optimization (QUBO) problem is a kind of optimization problem that can be transformed to the ground state search problem on the Ising model. Examples of combinatorial optimization problems that have been formulated as QUBO problems include the traveling salesperson problem [17] and the polyomino puzzle [16,21,22,23]. In particular, Takabatake et al. [23] have explored the generalized version of polyomino puzzles involving the use of various sizes of two-dimensional polyomino pieces, and three-dimensional polycubes. They also suggest its potential applications in drug discovery.

Research on applying the Ising machine to drug discovery has begun. Sakaguchi et al. [24] modeled a compound placement problem where all correct atom positions in the docking pose are precisely given and only atom type assignments are unknown. Banchi et al. [25] proposed a method for matching known interaction sites of proteins with compound structures. They utilized a Gaussian boson sampler to sample many initial solutions, followed by shrinking and expansion of the matching using classical computational methods. Their approach superimposed the rigid compound structure onto the protein surface. Zha et al. [26] performed structure matching for computationally estimated interaction points of proteins with compound structures. They matched the relative distances between the rigid compound’s atomic positions and the interaction points of the protein, followed by the superimposition of them. However, the problem setting of Sakaguchi et al. assumed that the relative positions of the protein and compound are already known. Banchi et al. utilized experimentally known interaction sites, requiring sufficient knowledge for the target protein. Zha et al. did not rely on knowledge about the target protein; however, they ignored the internal degrees of freedom of compounds, although they are essential for docking calculation. These three methods fall far short of realistic docking calculation used in drug discovery.

Therefore, in this study, we formulated protein–ligand flexible docking, which adequately considers the internal degrees of freedom of the compound, as a QUBO problem. First, we extracted the essence of an existing fragment-based docking strategy, which includes the enumeration of many candidate fragment placements and the selection of a set of placements that are consistent as the compound. In our formulation, each binary decision variable of the QUBO corresponds to a candidate fragment placement, which is enumerated. Subsequently, we designed a Hamiltonian of the problem with four terms representing four factors; (1) protein–fragment binding free energy ΔG, (2) penalty for clash between fragment placements, (3) reward for forming covalent bonds between fragment placements, and (4) constraints to ensure that only a single placement is chosen for each fragment. We also implemented a proof-of-concept system with the use of SQBM+, which is a simulated quantum annealer.

## 2. QUBO Problem Formulation of Fragment-Based Docking

### 2.1. Fundamental Factors of Fragment-Based Docking Calculation

We propose formulating fragment-based protein–ligand flexible docking as a QUBO problem. As a first step of the formulation, we extracted the fundamental factors of a fragment-based docking. A fragment-based docking tool, eHiTS [12] conducts maximum clique finding of fragment placement graph $G=\left(V,E\right)$, where each vertex $v_{i}$ corresponds to each fragment placement $p_{i}$. Note that a clique is a subset of vertices of an undirected graph such that every two distinct vertices in the clique are adjacent. The maximum clique finding problem is a kind of combinatorial optimization problems and we referred to the algorithm.

Rigid fragments $f_{1},f_{2},\ldots$ are decomposed from the compound structure of interest, and they are subsequently docked into a binding site of a target protein to obtain fragment placements $p_{i}$ ($v_{i}$ in the graph *G*). Edges $e_{ij}$ are added into the graph *G* if the fragment placements pair $p_{i},p_{j}$ satisfies all the following conditions.

1. Fragment $f_{i}$ of $p_{i}$ and fragment $f_{j}$ of $p_{j}$ are different.
2. $p_{i}$ and $p_{j}$ do not clash.
3. If $f_{i}$ and $f_{j}$ are connected in the compound structure of interest, the placements $p_{i},p_{j}$, can be connected in the same manner as the compound.

As a clique of the graph implies a consistent fragment placement set as the compound, this enables flexible docking with consideration of the internal degrees of freedom of the compound.

Therefore, the fundamental factors of the fragment-based docking calculation into the combinatorial optimization problem are the followings:

1. Decompose a compound into multiple fragments (chemical substructures with no internal degree of freedom) and regard chemical compound structure as a set of fragment placements.
2. Enumerate candidate fragment placements by independent fragment docking.
3. Choose a single placement for each fragment.
4. Consider clashes between fragments.
5. For fragments that have covalent bonds with each other, consider the bond distance between the placements.

### 2.2. Similarities and Differences between Polyomino Puzzle and Fragment-Based Docking

**Figure 1:**
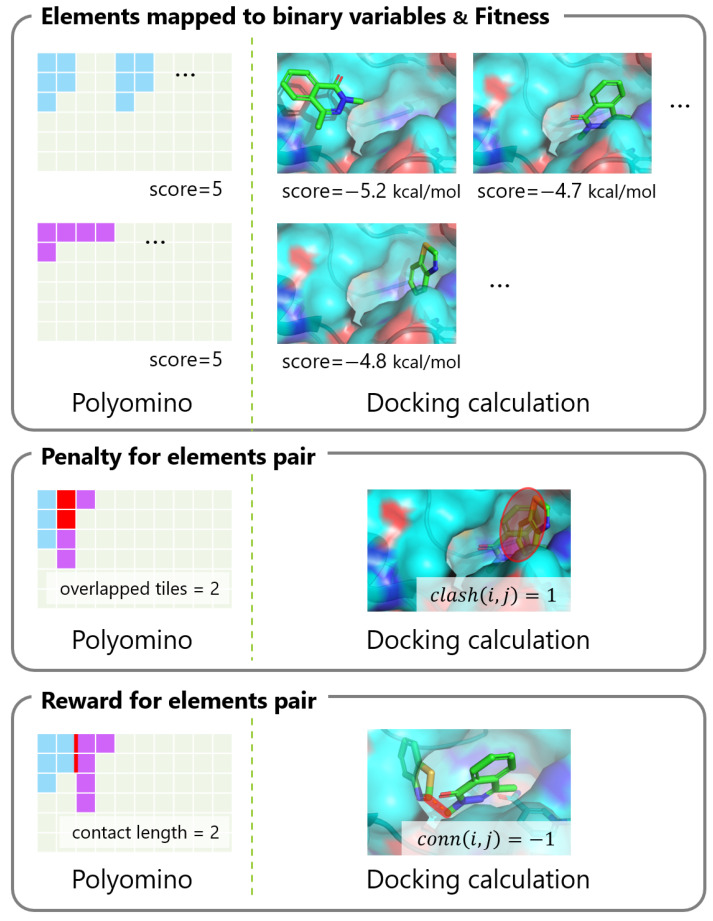
Comparing factors for QUBO problem formulation between polyomino puzzle and docking calculation.

In Section 2.1, we itemized the factors that should be considered when formulating a fragment-based docking calculation for the QUBO problem. Some of these factors have similarities to the Hamiltonian presented by Takabatake et al. [23] in their approximation of the protein–compound docking calculation as a generalized polyomino puzzle. Table 1 and Figure 1 show the comparisons between the polyomino puzzle and the docking calculation when modeled as a QUBO problem.

### 2.3. Elements to Be Mapped to Binary Variables

The elements of the problem that are mapped to variables are an important factor to be determined, as they can significantly change the difficulty of a combinatorial optimization problem. As already mentioned in Section 2.1, we assigned a single binary variable $x_{i}$ for each fragment placement in 3D space.

As the number of fragment placements in 3D space is innumerable, we needed to set some rules to limit their number. The simplest idea was to select fragment placements only with good fitness (binding free energy between the target protein and the fragment). However, fragment placements composing actual compound structures in protein–compound complex structures sometimes contain fragments that themselves do not have favorable binding free energies with the target protein. Such placements serve as linkers that connect other fragment placements which have good binding free energies [12].

### 2.4. Fitness for Each Variable

The fitness for the variables is the local gain by accepting the fragment placement. In fragment-based docking, the binding free energy score of a compound is expressed as the sum of the binding free energy scores of selected fragment placements. As the sum can be expressed by a first-order term, the energy score of a compound is described as:

(1) $$H_{1}=\sum_{i}\Delta G_{i}x_{i}$$

where $\Delta G_{i}$ is the binding free energy score between corresponding fragment placement $p_{i}$ and the target protein, and $x_{i}\left(\in \{0,1\}\right)$ is the binary decision variable whether to accept $p_{i}$ or not.

### 2.5. Penalty for Element Pair

Subsequently, we considered pairwise relationships between the elements. For instance, the optimal solution of the polyomino puzzle [23] requires that all polyominoes must not overlap each other. Similarly, the co-occurrence of fragment placements that clash with each other is inconsistent as a compound structure and should be penalized for such clashes. Therefore, we used clash(i,j)≥0 to express the penalty for clashes as follows.

(2) $$H_{2}=\sum_{i,j}\mathrm{clash}\left(i,j\right)x_{i}x_{j}$$

### 2.6. Reward for Element Pair

Unlike the clash between fragment placements discussed in Section 2.5, for the reconstruction of compound structures, the placements $p_{i},p_{j}$ of fragments $f_{i},f_{j}$ that are covalently linked must be arranged in a positional relationship such that covalent bonding is possible. Therefore, we used conn(i,j)≤0 to express the reward for the possible covalent bond as follows.

(3) $$H_{3}=\sum_{i,j}\mathrm{conn}\left(i,j\right)x_{i}x_{j}$$

### 2.7. Constraints for the Number of Selected Placements

As mentioned in the introduction, docking calculations estimate the binding pose between a target protein ${\mathrm{Prot}}_{m}$ and a chemical compound ${\mathrm{Cmpd}}_{n}$ in a compound library. For example, suppose ${\mathrm{Cmpd}}_{n}$ consists of three fragments F={a,b,c}. To reconstruct the compound structure, only a single placement of fragment *a* must be assigned. Likewise, only a single placement for each of the fragments *b* and *c* must be assigned. This is exactly the same as the condition “each polyomino is used once” in the polyomino puzzle; thus, the constraint regarding the number of selections can be written as follows:

(4) $$H_{4}=\frac{1}{2}\sum_{k}^{F}{\left(\sum_{f_{i}=k}x_{i}-1\right)}^{2}+\frac{1}{2}\sum_{k}^{F}\sum_{f_{i}=k}x_{i}\left(1-x_{i}\right)$$

where *F* is a set of fragments that compose the compound ${\mathrm{Cmpd}}_{n}$, and $f_{i}$ is a fragment of a placement $p_{i}$.

## 3. Materials and Methods

### 3.1. Method Overview

**Figure 2:**
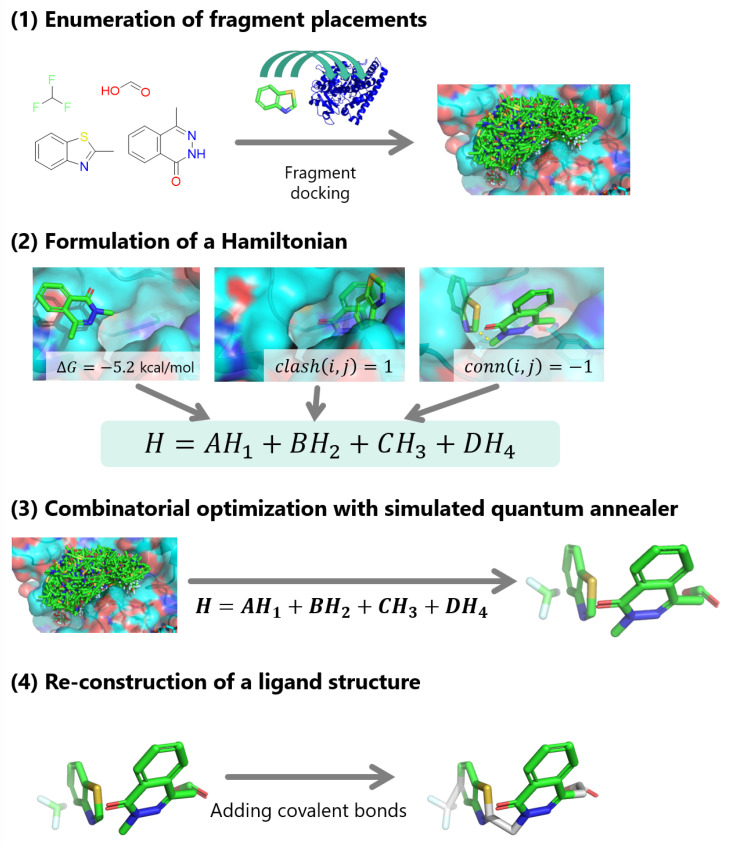
A workflow of the proof-of-concept implementation.

In Section 2, we described how the docking calculation should be formulated as a QUBO problem. The Hamiltonian was practically designed for flexible docking in this section as a proof-of-concept for this formulation as shown in (Figure 2): Step 1. enumeration of fragment placements, Step 2. formulation of a Hamiltonian, Step 3. combinatorial optimization with simulated quantum annealer, and Step 4. reconstruction of a compound structure.

### 3.2. Pose Enumeration with Protein–Fragment Rigid Docking

As discussed in Section 2.3, the fragment placements subject to combinatorial optimization must efficiently enumerate with good binding free energy scores while preserving positional diversity. Thus, the docking region was divided into multiple 2 Å × 2 Å × 2 Å subregions, and protein–fragment rigid docking was independently performed for each subregion after decomposing the compound into fragments. We used REstretto [11] as a docking tool. Several parameters are set to preserve placement diversity: local optimization of placement is not conducted (NO_LOCAL_OPT = True), placements having negative (good) energy scores are extracted (OUTPUT_SCORE_THRESHOLD = 0.0 kcal/mol), and each output placement has a root mean square deviation (RMSD) of at least 1.0 Å away from each other (POSE_RMSD = 1.0 Å). If the number of extracted placements is over 20, the best 20 placements per fragment are output (POSES_PER_LIG = 20). The placement output from different subregions may have similar placement (RMSD less than 1.0 Å). Only one placement with the best free energy score is retained from such a similar group.

### 3.3. QUBO Formulation

The Hamiltonian *H* (the objective function to be minimized) is formulated as follows, with the four terms described in Section 2:

(5) $$\begin{array}{c} H=AH_{1}+BH_{2}+CH_{3}+DH_{4} \end{array}$$

where coefficients A,B,C,D(≥0) are adjustable constants that determine the contribution weights of each term. Since the $H_{2}$ and $H_{3}$ terms are both related to the pairwise relationship with other fragment placements, their constants *B* and *C* should be of a similar scale.

Furthermore, since the $H_{4}$ term is a constraint term whose condition must be satisfied, the weight *D* should be larger. In this study, the weights of each term were determined as (A,B,C,D)=(1,5,5,25) according to a prior experiment based on the aforementioned assumptions, with the constant A=1 fixed.

### 3.4. Criteria of Covalent Bonding and Collision

The functions $\mathrm{clash}(p_{i},p_{j})$ involved in the $H_{2}$, which represents a clash, and $\mathrm{conn}(p_{i},p_{j})$ involved in $H_{3}$, which represents a covalent bond, are calculated based on physico-chemical parameters: bond length, bond angle, dihedral angle of the covalent bond, and inter-atomic distance between each atom. The interaction energies can be calculated with a force field [27,28,29]. However, it is almost impossible to obtain placements pairs that have precisely appropriate bond lengths, bond angles, and dihedral angles for $\mathrm{conn}(p_{i},p_{j})$, owing to the limited resolution of fragment placements in this study. Therefore, distortion energy of a covalent bond and inter-molecular repulsion should be calculated tolerantly for errors. Thus, we dare to employ binary functions for $\mathrm{clash}(p_{i},p_{j})$ and $\mathrm{conn}(p_{i},p_{j})$, representing whether the condition is applicable or not.

1. Calculate interaction energy $E_{\mathrm{NB}}\left(p_{i},p_{j}\right)$ for each fragment placements pair $p_{i},p_{j}$ without adding a covalent bond.
2. Calculate interaction energy $E_{B}\left(p_{i},p_{j}\right)$ for each fragment placement pair $p_{i},p_{j}$ with the addition of a covalent bond if the fragments $f_{i}$ and $f_{j}$ have a covalent bond.
3. Set conn(i,j)=−1 if the fragments $f_{i}$ and $f_{j}$ have a covalent bond and $E_{B}\left(p_{i},p_{j}\right)\leq {\mathrm{th}}_{E}$; otherwise, set conn(i,j)=0. Note that ${\mathrm{th}}_{E}$ is an energy tolerance level.
4. Set clash(i,j)=1 if conn(i,j)=0 and $E_{\mathrm{NB}}\left(p_{i},p_{j}\right)>{\mathrm{th}}_{E}$; otherwise, set clash(i,j)=0.

Interaction energies $E_{\mathrm{NB}}\left(p_{i},p_{j}\right)$ and $E_{B}\left(p_{i},p_{j}\right)$, based on the Universal Force Field (UFF) [27], are calculated using RDKit (version 2023.09.5). Note that $E_{B}\left(p_{i},p_{j}\right)$ includes distortion energy from the added covalent bond and non-bond interaction energy between atoms within the single molecule structure composed of the two fragments and the added covalent bond. The distortion energy originates from the bond length, bond angle, and dihedral. To accept some distortions and clashes, the energy tolerance level ${\mathrm{th}}_{E}$ is set to a high value of 500 kcal/mol.

### 3.5. Combinatorial Optimization by SQBM+

Solutions of the QUBO problem formulated in Section 3.2, Section 3.3 and Section 3.4 are obtained by using SQBM+, a simulated quantum annealer. SQBM+ outputs many local solutions, which is suitable for docking calculation as the docking is expected to output several docked compound poses.

SQBM+ is a quantum-inspired optimization solution based on the Simulated Bifurcation Machine (SBM), which is a combinatorial optimization solver utilizing the simulated bifurcation algorithm (SB algorithm) [20]. SQBM+ has novel exploration algorithms such as classical adiabatic exploration and ergodic exploration, and it can handle large-scale QUBO problems with 10 million variables. Some extensions, such as the support of polynomial unconstrained binary optimization (PUBO) with cubic and quartic problems and enabling continuous variables were performed with the capabilities of the SB algorithm [30]. An example of an application is a high-speed automated trading system in finance [31]. The detection of trading opportunities in an extended pair-trading strategy was formulated as an optimal path-search problem in a directed graph, and solutions were obtained by the SB algorithm. Another example of an application is the automotive computing platform in the mobility industry [32].

We used SQBM+ for AWS (version 2.0.1) and collected and analyzed local solutions obtained in 300 s of computation (timeout=300).

### 3.6. Postprocessing

The local solutions output by SQBM+ may have selected two or more placements for one fragment $f_{i}$. Therefore, only the solutions containing a single placement for each fragment composing the compound are extracted by a postprocessing filter program from all the obtained solutions.

### 3.7. Dataset Preparation

We conducted a redocking experiment to evaluate the performance of the proof-of-concept implementation. In the redocking experiment, a compound of an experimentally known complex structure with a drug target protein was extracted and docked to the protein structure again. It is generally regarded as an “acceptable” redocking pose if the RMSD between a predicted binding pose and the experimentally known binding pose is less than RMSD=2.5 Å [33] without the use of compound 3D structural information of the bound state.

Here, we conducted a redocking experiment with aldose reductase (ALDR). ALDR protein is composed of approximately 300 amino acids and is an enzyme catalyzing the reduction of glucose to sorbitol by nicotinamide adenine dinucleotide phosphate (NADPH). It is thought to be associated with neuropathy in diabetes, and thus, it is a drug target protein. An approved drug called epalrestat inhibits this protein.

**Figure 3:**
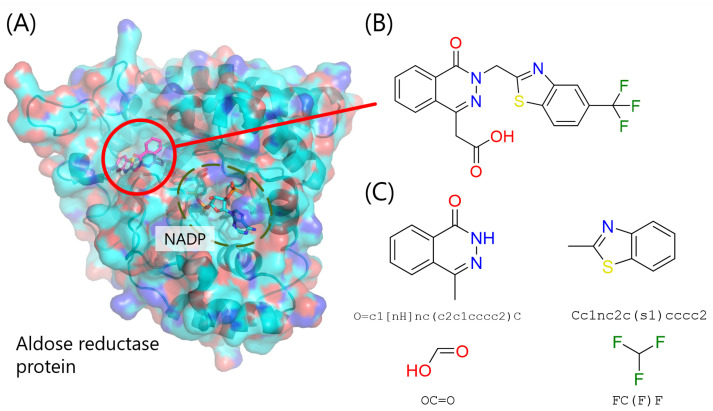
The complex structure of aldose reductase (ALDR) and its inhibitor. (**A**) A molecular complex formed by the target protein coupled to small molecule: the cofactor NADP and inhibitor. The protein is represented by a molecular surface with the backbone atoms traced, and the cofactor NADP and inhibitor are shown as sticks. Proteins and small molecules are shown in colors based on the element of the atoms: the oxygen and nitrogen atoms are colored red and blue, respectively; the carbon atoms of the protein and cofactor are indicated in blue; and the carbon atoms of the inhibitor are indicated in purple. (**B**) Structural formula of the inhibitor compound. (**C**) Fragments decomposed from the inhibitor with SMILES (simplified molecular input line entry system), which is a linear notation for describing the structural formula of compounds.

First, co-crystallized 3D structure of ALDR and its known inhibitor compound was obtained from Protein Data Bank (PDB) [34,35] (Figure 3). Subsequently, the inhibitor compound was decomposed into fragments based on the compound decomposition algorithm of Spresso [36]. The ionization states and 3D structure of all fragments were re-generated with LigPrep (Schrödinger, Inc., New York, NY, USA). Then, the docking region for fragment docking (Section 3.2) was determined as shown in Table 2. The box center was the center of the co-crystallized compound 3D structure, and docking region was manually determined to cover the entire area involving the binding site of the co-crystallized compound.

## 4. Results and Discussion

### 4.1. Fragment Docking

**Figure 4:**
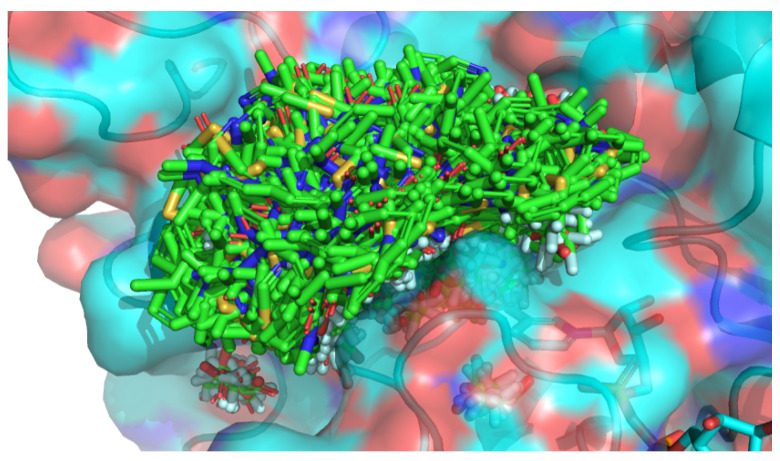
The result of fragment docking. All candidate fragment placements and protein 3D structures are shown in green and cyan, respectively.

Overall, 3005 candidate fragment placements were obtained by fragment docking based on the procedure described in Section 3.2. All fragment placements are superimposed in Figure 4. Fragment placements were spread over the entire pocket space of the ALDR owing to the division of the binding pocket region into 343 subregions.

**Figure 5:**
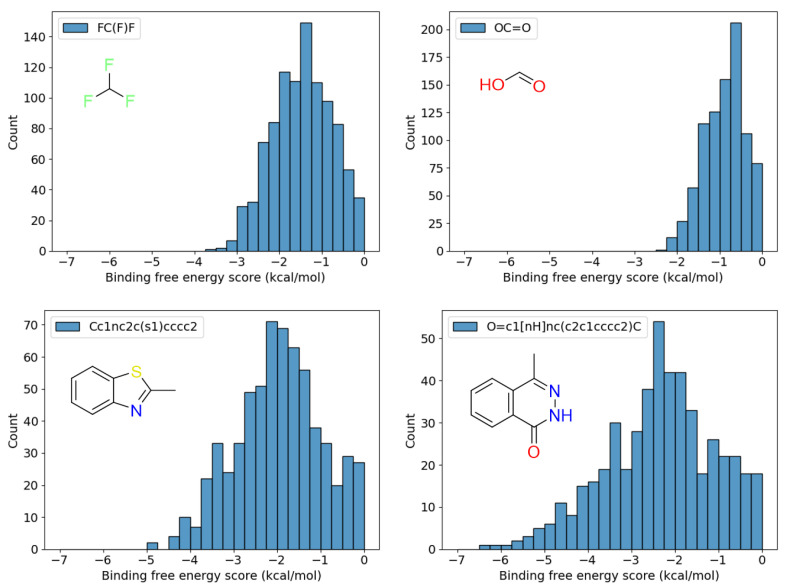
Histograms of binding free energy scores for each of the four constituent fragments.

Table 3 shows the number of candidate fragment placements obtained for each fragment, as well as the minimum (best) and maximum (worst) values of their binding free energy scores. Figure 5 shows the distribution of binding free energy scores of all fragments. Notably, the placements with less favorable and favorable binding free energy scores remain as candidates. By obtaining fragment placements for each subregion with good scores, we could obtain fragment placements that can act as linkers in regions that have little interaction with the protein, suggesting that positional diversity of placements was preserved.

### 4.2. Local Solutions Enumerated by SQBM+

**Figure 6:**
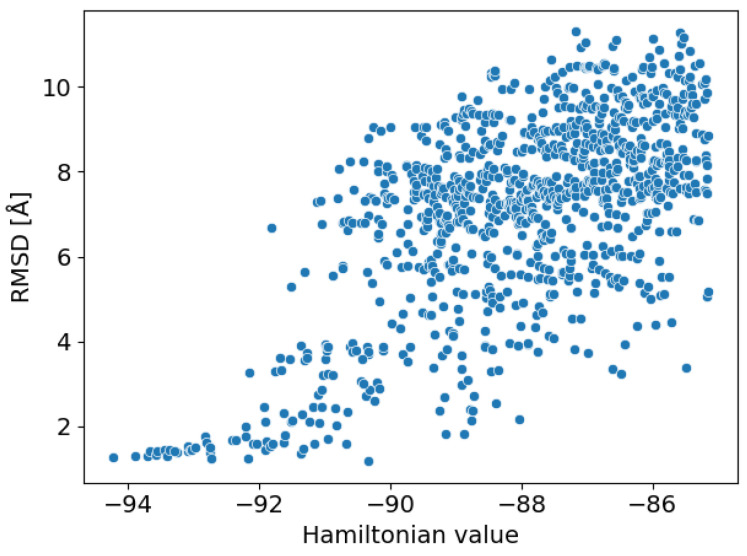
The scatter plot of the Hamiltonian values and the RMSD with corresponding docked poses to the co-crystallized compound structure. Only the top 1000 local solutions are shown.

We obtained 7298 local solutions as outputs of SQBM+. After postprocessing, there were 5414 (74.2%) effective solutions wherein the placements of all fragments were selected once each. Figure 6 shows the scatter plot of the Hamiltonian values and the RMSD with corresponding docked poses to the co-crystallized compound structure. A funnel-shape was observed where the lower the Hamiltonian value (corresponding to the better docking score with less structural inconsistency of a compound), the lower the RMSD value, indicating that the Hamiltonian was appropriately designed.

**Figure 7:**
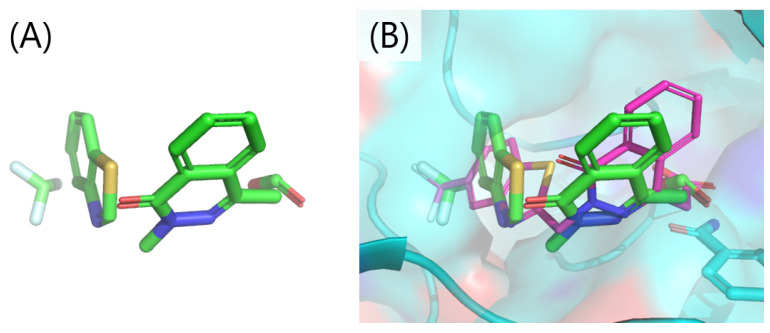
The fragment placement set translated from the best solution. (**A**) the fragment placement set. (**B**) comparison between the fragment placement set and the co-crystallized structure. The fragments and co-crystallized structure are shown as sticks, and the protein is represented by a molecular surface with the cartoon representation. Small molecules and protein are shown in colors based on the element of the atoms: the oxygen and nitrogen atoms are colored red and blue, respectively; the carbon atoms of the fragments are indicated in green; the carbon atoms of the co-crystallized structure are indicated in purple; and the carbon atoms of the protein are indicated in blue.

Figure 7 shows the fragment placement set interpreted from the best solution in terms of Hamiltonian value. All fragments were located close to each other, and it seems consistent as a compound structure (Figure 7A). The predicted binding pose that was reconstructed from the set of fragment placements is accurate enough since the RMSD was 1.26 Å with the co-crystallized structure (Figure 7B).

### 4.3. Energy Minimization of Reconstructed Compound Structure

**Figure 8:**
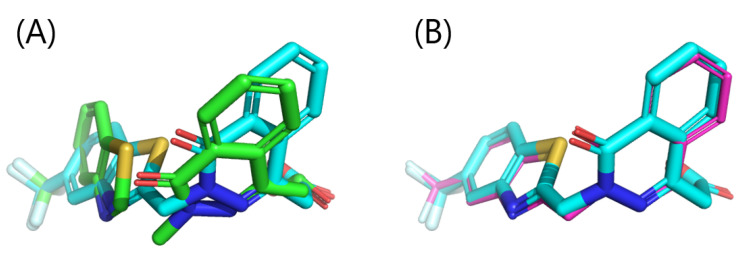
The compound structure after energy minimization. (**A**) Comparing the structure before and after energy minimization. (**B**) Comparing the structure after energy minimization with the co-crystallized structure. The fragments and small molecules are shown as sticks in colors based on the element of the atoms: the oxygen and nitrogen atoms are colored red and blue, respectively; the carbon atoms of the structure before energy minimization are indicated in green; the carbon atoms of the structure after energy minimization are indicated in cyan; and the carbon atoms of the co-crystallized structure in purple.

The structure shown in Figure 7A has left structural distortion, and it is inappropriate to refer to it as final estimation of the binding pose. Therefore, the reconstructed compound structure was optimized in the rigid protein 3D structure using energy minimization of the compound (which is widely applied manner to eliminate structural distortion) with Maestro (Schrödinger, Inc., version 2020-2). First, the predicted complex structure composed of the 3D structure of ALDR (PDB ID: 2HV5) and reconstructed compound structure was made. “Preprocess” and “H-bond assignment” in Protein Preparation Wizard was applied, followed by the energy minimization of compound structure in the OPLS3e force field [29] with proteins and cofactors regarded as rigid bodies. Consequently, the RMSD between the minimized structure and the co-crystallized structure was 0.27 Å (Figure 8), which is almost the identical structure.

### 4.4. Toward Virtual Screening Applications

As the current implementation intends to be a proof-of-concept, the calculation remains inefficient. To obtain the results, we spent approximately 100 CPU core min (Intel Core i7-9700) for exhaustive fragment docking of four fragments, approximately 40 CPU core min (Intel Core i7-9700) for calculation of interaction energies between fragment placements, and 5 min for SQBM+, resulting in calculations of more than 2 CPU core hours in total. The inefficiencies include the redundant pre-calculation of fragment docking because a number of docking calculations for all subregions are conducted independent each other, resulting in a 10–100 times larger calculation cost. Additionally, the interaction energies $E_{B}$ and $E_{\mathrm{NB}}$ were calculated even for far distant fragment pose pairs for which conn(i,j)=0 and clash(i,j)=0 obviously in the current implementation. With improvement of the implementation, we estimate that the docking can be performed within about ten minutes. Approximately 28 million compounds are composed of only approximately 260 thousand fragments [36]; thus, the computational cost for the exhaustive fragment docking as the first step in the process will become negligible in large-scale virtual screening.

Nevertheless, further accelerations are required to apply this approach to practical virtual screening, which requires evaluating more than millions of compounds. To meet the requirement, we are working toward proposing a novel strategy to select multiple compounds in a single combinatorial optimization. In this case, three issues arise: (i) the number of types of fragments is huge, (ii) therefore, it must be considered as a typical case that any placement of a given candidate fragment is not chosen, and (iii) atoms that constitute a covalent bond are difficult to identify beforehand. In particular, as for issue (i), it is impractical to consider all placements of hundreds of thousands of possible fragments as candidates for combinatorial optimization. Therefore, we are working on a method to represent numerous fragments with a small number of representative fragments based on structural and functional similarity and devising a plan to estimate feasible compounds from the optimization results of representative fragment placements.

## 5. Conclusions

In this study, we formulated fragment-based protein–ligand flexible docking as a QUBO problem. We designed a Hamiltonian of the problem with four terms; (1) protein–fragment binding free energy ΔG, (2) penalty for clash between fragment placements, (3) reward for forming covalent bonds between fragment placements, (4) constraints to ensure that a single placement is chosen for each fragment. It should be highlighted that it is the first formulation to treat internal degrees of freedom of compound. The redocking experiment with Aldose reductase (ALDR) and its inhibitor showed that the proof-of-concept implementation could obtain accurate binding pose prediction. Energy minimization of the predicted compound structure further improved the accuracy of the structure compound structure.

The implementation is a proof-of-concept, and future improvement of the calculation efficiency is mandatory. For instance, distance-based determination of clash(i,j) and conn(i,j) is a possible selection. However, the proposed strategy is expected to be more exhaustive than the heuristic pose search methods employed by conventional docking tools, since it performs combinatorial optimization from among thousands of candidates. In particular, it may be expanded to docking of flexible molecules (such as peptides), for which conventional docking tools have low prediction accuracy owing to insufficient conformational search and docking calculations that consider the flexibility of protein side chains.

## Figures and Tables

**Table 1:** Comparison between the polyomino puzzle and the docking calculation as a QUBO problem.

	Polyomino Puzzle	Docking Calculation
**Elements mapped to binary variables**	placements of polyominos on the board	placements of fragments
		in the protein pocket
**Fitness for each element**	sizes of polyominos	binding free energy scores
		to the protein
**Penalty for elements pair**	overlaps between polyomino placements	clashes between fragment placements
**Reward for elements pair**	length of touching borders	chemical bond between
	between placements	fragment placements
**Constraints for the number**	a single placement per one polyomino	a single placement per one fragment
**of selected elements**		

**Table 2:** Target protein information and parameters of fragment docking.

Target	Aldose reductase (ALDR)
PDB ID	2HV5
Box center	(16.61 Å, −7.03 Å, 14.45 Å)
Volume of the docking region	14 Å × 14 Å × 14 Å
The number of subregions	$343\;(=7^{3})$ subregions of 2 Å × 2 Å × 2 Å

**Table 3:** The result of fragment docking of each fragment.

Fragment SMILES	Number of Poses	Binding Energy Range (kcal/mol)
FC(F)F	982	−3.592–−0.008
OC=O	884	−2.420–−0.001
Cc1nc2c(s1)cccc2	641	−4.983–−0.009
O=c1[nH]nc(c2c1cccc2)C	498	−6.288–−0.039

## Data Availability

The complex structure of ALDR and its inhibitor can be obtained from the Protein Data Bank (PDBID: 2HV5). An SDF file containing top 1000 docked compound structure with the Hamiltonian values has been deposited in Zenodo (https://doi.org/10.5281/zenodo.10889782, accessed on 28 April 2024). REstretto is available at https://github.com/akiyamalab/restretto/, accessed on 28 April 2024.

## Abbreviations

The following abbreviations are used in this manuscript:

ALDR	Aldose Reductase
FPGA	Field-Programmable Gate Array
GPU	Graphics Processing Unit
OPLS	Optimized Potentials for Liquid Simulations
PDB	Protein Data Bank
QUBO	Quadratic Unconstrained Binary Optimization
RMSD	Root Mean Square Deviation
SB	Simulated Bifurcation
SBM	Simulated Bifurcation Machine
SMILES	Simplified Molecular Input Line Entry System
UFF	Universal Force Field
